# Ruthenium Dendrimers against Human Lymphoblastic Leukemia 1301 Cells

**DOI:** 10.3390/ijms21114119

**Published:** 2020-06-09

**Authors:** Sylwia Michlewska, Maksim Ionov, Aleksandra Szwed, Aneta Rogalska, Natalia Sanz del Olmo, Paula Ortega, Marta Denel, Damian Jacenik, Dzmitry Shcharbin, Francisco Javier de la Mata, Maria Bryszewska

**Affiliations:** 1 Laboratory of Microscopic Imaging & Specialized Biological Techniques, Faculty of Biology & Environmental Protection, University of Lodz, 90-237 Lodz, Poland; 2 Department of General Biophysics, Faculty of Biology & Environmental Protection, University of Lodz, 90-236 Lodz, Poland; aleksandra.szwed@biol.uni.lodz.pl (A.S.); maria.bryszewska@biol.uni.lodz.pl (M.B.); 3 Department of Medical Biophysics, Faculty of Biology & Protection, University of Lodz, 90-236 Lodz, Poland; aneta.rogalska@biol.uni.lodz.pl (A.R.); martadenel@gmail.com (M.D.); 4 Networking Research Center on Bioengineering, Biomaterials & Nanomedicine (CIBER-BBN), 28029 Madrid, Spain; n.sanzdelolmo@gmail.com (N.S.d.O.); paula.ortega@uah.es (P.O.); javier.delamata@uah.es (F.J.d.l.M.); 5 Department of Organic Chemistry and Inorganic Chemistry, Research Institute of Chemistry “Andrés M. del Rio (IQAR)”, Institute “Ramón y Cajal” for Health Research (IRYCIS), University of Alcalá, 28805 Madrid, Spain; 6 Department of Cytobiochemistry, Faculty of Biology & Protection, University of Lodz, 90-236 Lodz, Poland; damian.jacenik@biol.uni.lodz.pl; 7 Institute of Biophysics & Cell Engineering of NASB, 220072 Minsk, Belarus; shcharbin@gmail.com

**Keywords:** 1301 human lymphoblastic leukemia, carbosilane dendrimer, ruthenium, drug delivery, cytotoxicity

## Abstract

Ruthenium atoms located in the surfaces of carbosilane dendrimers markedly increase their anti-tumor properties. Carbosilane dendrimers have been widely studied as carriers of drugs and genes owing to such characteristic features as monodispersity, stability, and multivalence. The presence of ruthenium in the dendrimer structure enhances their successful use in anti-cancer therapy. In this paper, the activity of dendrimers of generation 1 and 2 against 1301 cells was evaluated using Transmission Electron Microscopy, comet assay and Real Time PCR techniques. Additionally, the level of reactive oxygen species (ROS) and changes of mitochondrial potential values were assessed. The results of the present study show that ruthenium dendrimers significantly decrease the viability of leukemia cells (1301) but show low toxicity to non-cancer cells (peripheral blood mononuclear cells—PBMCs). The in vitro test results indicate that the dendrimers injure the 1301 leukemia cells via the apoptosis pathway.

## 1. Introduction

Currently, among all blood cancers—acute myeloid leukemia (AML), acute lymphoblastic leukemia (ALL), chronic myeloid leukemia (CML), and chronic lymphocytic leukemia (CLL) [1,2]—treatment of ALL is the most effective. The survival rate of patients with this type of cancer has increased significantly, especially among children. However, despite wide knowledge and use of steroid and chemotherapeutic treatments of cancers, many ALL patients are not yet cured [3,4]. This remains the main cause of mortality, notably among children [5]. Moreover, leukemia belongs to the group of cancers that develop secondary resistance to chemotherapy. In recent years, many publications have explored the increased expression of glycoprotein P (Pgp), which is considered an unfavorable prognosis for leukemia patients [6]. Further, overexpression of Mcl-1, Bcl-xL, and Bcl-2, members of the anti-apoptotic protein family, is responsible for cancer cell resistance to chemotherapy [7,8,9].

Even though there are many effective cancer therapies [10], the new generation of drugs still cannot guarantee successful treatment. Numerous side effects and possible complications stimulate the search for new and more effective ways of increasing the efficacy of chemotherapeutics and limiting the harm potentially resulting from them [3].

Nowadays, many researchers focus on designing effective anti-cancer therapies based on the delivery of genes and drugs by safe and effective nanocarriers. In current medical nanotechnology, dendrimers that can contain metal atoms at different points in their structure are important. Dendrimers are highly branched and monodisperse spherical molecules with different terminal surface groups that determine their physicochemical properties [11,12,13,14]. Dendrimers can bind genes and drug molecules and transport them to target cells, where they can be released. Metals with anti-cancer properties such as gold, silver, platinum, or ruthenium can be coordinated with the terminal surface groups [11,13,15]. Ruthenium seems particularly interesting because it can exist in various oxidation states, among which Ru(II) is strongly cytotoxic [11,16]. In addition, ruthenium resembles iron and can be similarly attached to transferrins and albumins [17,18]. Because of their rapid metabolism and increased demand for iron, cancer cells can take up ruthenium instead of iron [17,18]. Moreover, ruthenium atoms can perturb DNA replication in tumor cells, leading to cell death [16,17,18,19,20].

In the present study, carbosilane dendrimers functionalized with ruthenium (II) complexes in their periphery (CRD) were evaluated. The presence of this metal in the dendritic skeleton can make the resulting system toxic towards cancer cells but with lower toxicity towards normal cells. Such dendrimers can also be used as gene and drug carriers. All these factors make CRDs promising candidates for drug delivery in anti-cancer therapy.

The objective of this work is to unravel the mechanism of the anticancer activity of this family of first and second generation metallodendrimers, using the human ALL cell line 1301 as an example.

## 2. Results

### 2.1. Viability

**Figure 1:**
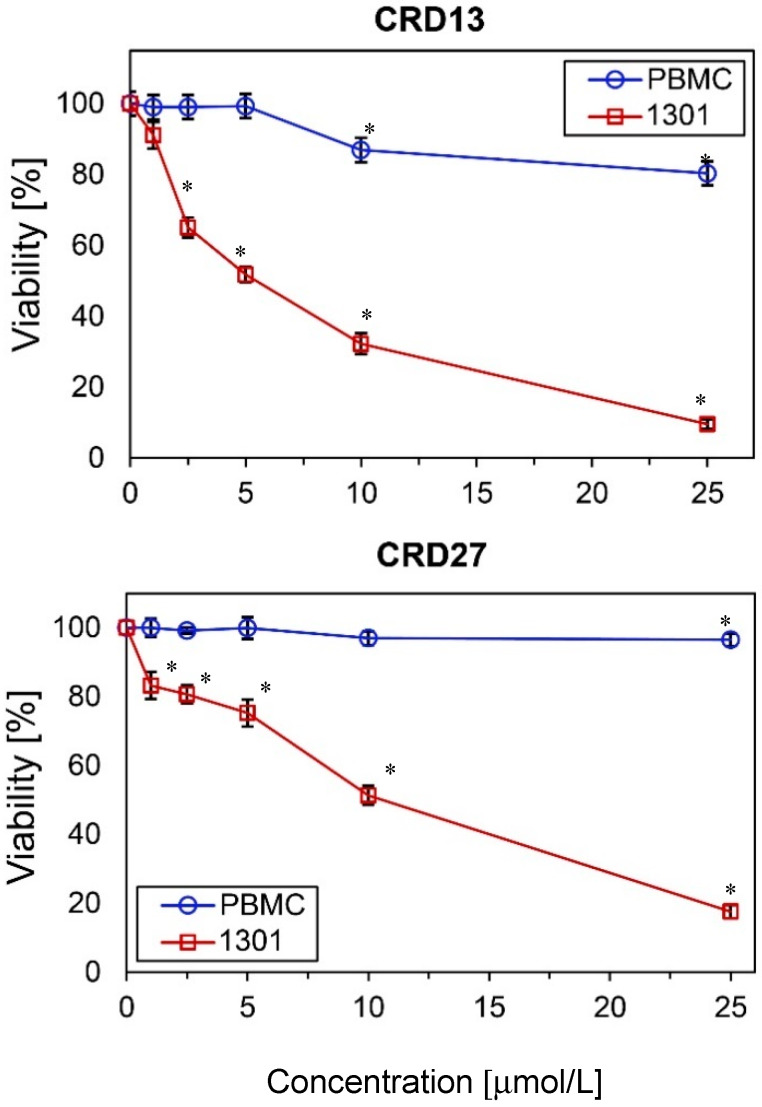
The effect of ruthenium-terminated carbosilane dendrimers (CRD) on viability of peripheral blood mononuclear cells (PBMCs) and 1301 cells after 24 h treatment. The values are mean ± SD of *n* ˃ 6. * Statistically significant differences compared to the control cells (* *p* < 0.05).

The cytotoxicity of the new carbosilane ruthenium metallodendrimers CRD13 and CRD27 towards cancer cells (1301 leukemia) and normal cells (peripheral blood mononuclear cells, PBMCs) was determined after 24 h incubation. The results show that viability depends on the dendrimer concentration. The number of living 1301 cells decreased significantly when 5 µmol/L CRD13 or 10 µmol/L CRD27 was present in the cell suspension (Figure 1). However, neither dendrimer at the same concentration had a cytotoxic effect on the (normal) PBMC cell suspension. The IC_50_ values for the 1301 cells after 24 h incubation were 3.80 ± 0.36 µmol/L for CRD13 and 4.9 ± 1.5 µmol/L for CRD27, and after 48 h were 3.7 ± 2.9 µmol/L for CRD13 and 3.8 ± 2.7 µmol/L for CRD27 (Table 1).

### 2.2. Transmission Electron Microscopy assay

#### 2.2.1. Light Microscopy of Semi-Thin Sections

**Figure 2:**
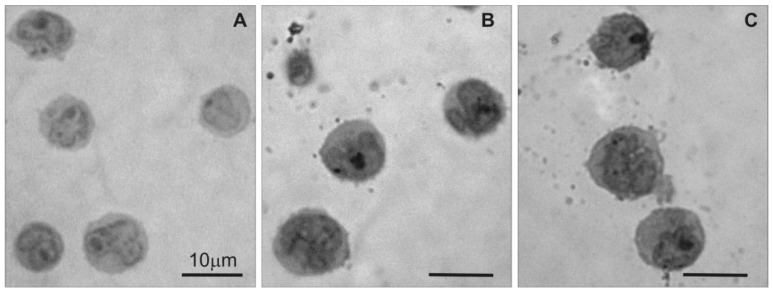
Light microscopy of semi-thin sections of 1301 cells: (**A**) untreated cells, (**B**) cells treated with CRD13 dendrimer at 2.5 µmol/L, (**C**) cells treated with CRD27 dendrimer at 5 µmol/L. Scale bar = 10 µm.

Light microscopy was used to examine changes in 1301 cell morphology in the semi-thin sections after treatment with CRD dendrimers. The image in Figure 2A shows that untreated 1301 cells exhibited standard morphology, but the CRD13 and CRD27 dendrimers caused changes (Figure 2B,C): the cells had denser cytoplasm and fewer surface vesicular structures.

#### 2.2.2. Transmission Electron Microscopy (TEM) of Ultra-Thin Sections

**Figure 3:**
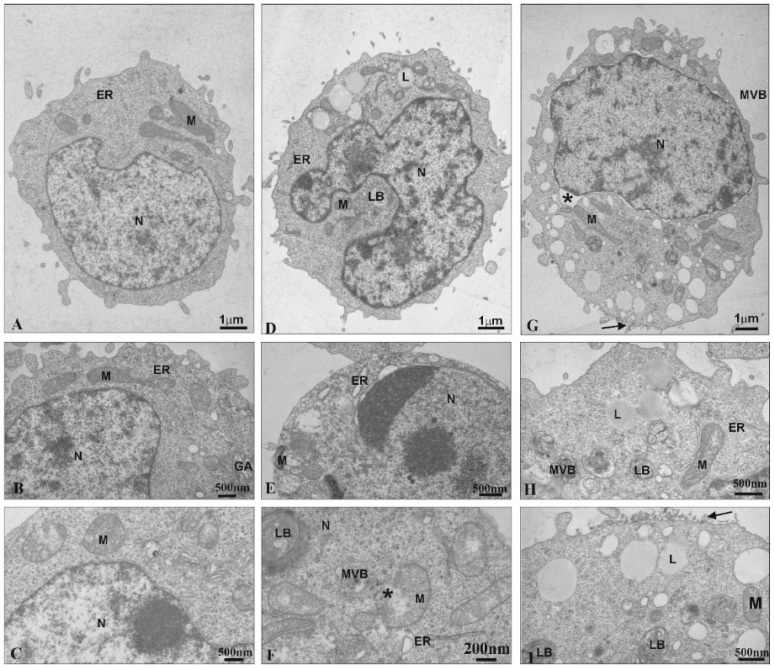
Ultrastructural changes in 1301 cells (**A**–**C**). Cells influenced by CRD13 at 2.5 µmol/L (**D**–**F**) or CRD27 at 5 µmol/L (**G**–**I**). Abbreviations: N—nucleus. Nu—nucleolus. M—mitochondria. MVB—multi-vesicular bodies. LB—lamellar bodies. L—lipids. GA—Golgi apparatus. ER—endoplasmic reticulum. *—vesicle containing CRD dendrimers.

Transmission Electron Microscopy was used to observe ultrastructural changes in 1301 cells under the influence of the CDR13 and CDR27 dendrimers. Untreated cells exhibited normal ultrastructure, including numerous microvilli on the surface, evenly distributed chromatin and numerous mitochondria (Figure 3A). Treatment with CRD13 changed the ultrastructure. The cell shape was slightly modified (Figure 3D). Chromatin marginalization and condensation were observed (Figure 3E). Moreover, multi-vesicular bodies, lamellar bodies, and numerous lipid structures were present. Additionally, the mitochondria contained small vacuoles, were swollen, and their membranes were damaged (Figure 3F).

After the cells were treated with CRD27, the nuclear envelopes were disorganized. Furthermore, the nuclear lamina separated from the chromatin, and multi-vesicular bodies and lamellar bodies appeared along with vacuoles in the mitochondria and numerous lipid deposits (Figure 3G–I). Additionally, secondary lysosomes were formed (Figure 3G,I) and electron dense granular material (probably dendrimers) appeared on the cell surfaces (Figure 3I).

### 2.3. Comet Assay

**Figure 4:**
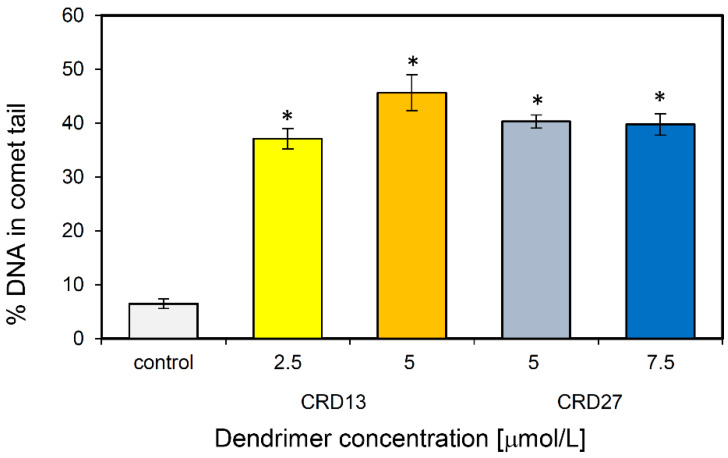
DNA (%) in the comet tail of the control cells and of 1301 cells treated with different concentrations of CRD13 and CRD27 dendrimers. Fifty cells were analyzed in each treatment. The values are the mean ± SD of three independent experiments. *Statistically significant differences from the control cells (* *p* < 0.05).

Comet assays were used to assess the ability of CRD13 and CRD27 to induce DNA damage. The results demonstrate that 1301 cells treated with CRD13 dendrimer for 24 h had more basal DNA damage at both concentrations tested than cells treated with CRD27 dendrimer (Figure 4). The highest level of DNA fragmentation (45.64 ± 3.32%) occurred when the cells were treated CRD13 at 5 µmol/L. The effect of CRD27 at both concentrations tested was also significant, when DNA fragmentation reached 40% (Figure 4).

### 2.4. Reactive Oxygen Species (ROS) and Mitochondrial Membrane Potential (ΔΨm)

The results in Table 2 show that treatment of 1301 cells with CRD13 and CRD27 dendrimers at 0.5–5.0 μmol/L for 0.5, 3, 24, and 48 h increased the ROS level and modified the mitochondrial potential (Table 2 and Table 3). Both studied dendrimers increased the ROS level in 1301 cells after 30 min of incubation by up to 140%. This effect was concentration-dependent, but during the following hours of treatment, this parameter decreased to the control values (Table 2).

The membrane potential in cells treated with CRD13 (30 min incubation) rose initially to 120% vs. control, and subsequently (3 h incubation) to 140%. Over the same time, the mitochondrial membrane potential had decreased to control values (Table 3). Incubation with the lower concentration of CRD27 for 24 or 48 h decreased the mitochondrial membrane potential, while higher concentrations increased this parameter up to 180% vs. control (Table 3); after 48 h incubation, this value returned to control level (Table 3).

### 2.5. Real Time PCR Changes in Expression of Pro- and Anti-Apoptotic Proteins

**Figure 5:**
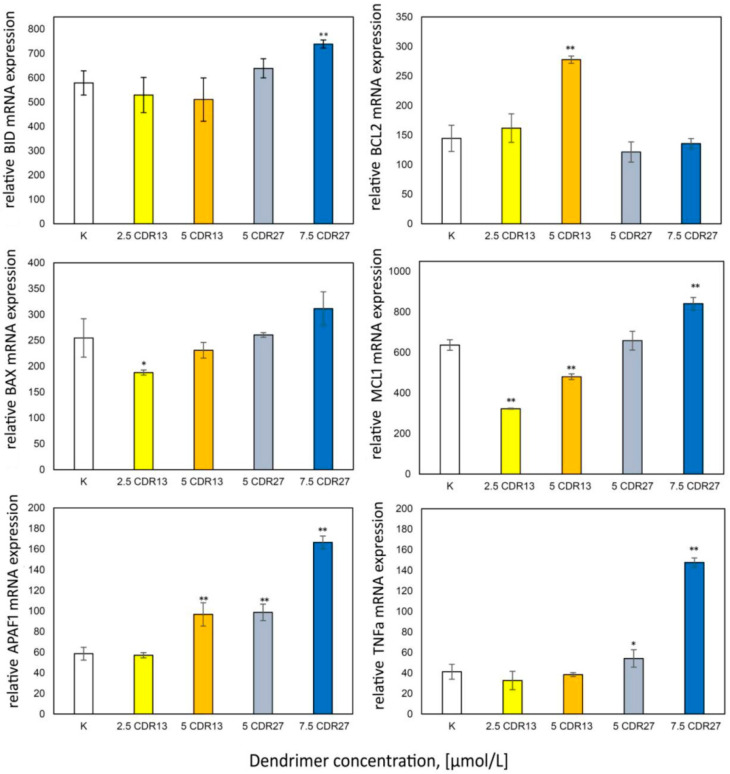
Relative anti-apoptotic protein expression values after 24 h incubation of 1301 cells with CRD13 and CDR27 at different concentrations. * Statistically significant differences from control cells (* *p* < 0.05, ** *p* < 0.01).

Apoptosis is regulated by Bcl-2 protein family members. Therefore, the expression of pro- and anti-apoptotic proteins was analyzed (Figure 5). The results show decreased expression of Mcl-1 and BAX in 1301 cells after incubation with low concentrations of CRD13 dendrimer; higher concentrations led to increases in Mcl-1 and BAX expression to values close to those in control cells. The Bcl-2 and APAF1 levels also increased. In cells treated with CRD27, TNFα, APAF1, BAX, BID, and Mcl-1 were expressed, each to a level higher than the value when the dendrimer concentration was 7.5 μmol/L.

## 3. Discussion

Dendrimers are known as the most promising delivery agents among all nanoparticles for treating numerous serious illnesses such as neurodegenerative diseases [15] or cancers [21,22,23]. They are also suggested as promising agents for diagnostic imaging in cancer therapy [10,21,22]. Ruthenium is well known as an anti-cancer metal [24,25], and carbosilane dendrimers that contain ruthenium on their surface have strong cytotoxic effects on different cancer cell lines [15]. The data obtained in this research show again that carbosilane metallodendrimers based on arene ruthenium (II) complexes, CRD13 and CRD27, at 5 µmol/L and 10 µmol/L are strongly cytotoxic to 1301 cells after 24 h incubation, but have low toxicity against normal cells. This result is consistent with those found by Pędziwiatr-Werbicka et al. for Hippo-18 cells and by Milowska et al. for PBMC cells [26,27]. The IC_50_ results indicate that both systems have very similar toxicity. However, at 10 µmol/L, the second generation CDR27 dendrimer produces higher cell viability than the first generation CDR13. A similar effect was described previously for 1301 and HL-60 cells [11,22].

The study of 1301 cells by TEM revealed that after treatment with CRD13 and CRD27 dendrimers, the cell morphology and structure changed, the cytoplasm became more dense, and the number of vesicular structures on the cell surface increased. In addition, electron dense granular material (probably dendrimers) was detected on the cell surface. According to literature data, nanoparticles can be taken up via macropinocytosis and clathrin-dependent endocytosis [28,29,30], and CRD27 was taken up by endocytosis in HL-60 cells [31]. The microimages of 1301 cells obtained in the present work show that CRD13 changed the cell ultrastructure: there were numerous multi-vesicular bodies, lamellar bodies, lipid deposits, and swollen mitochondria with small vacuoles. These results are in accordance with those previously reported for HL-60 cells treated with CRD13, which showed distinct chromatin condensation with changes in mitochondrial shape and the appearance of multivesicular and lamellar bodies [31], typical features of early apoptosis [32]. Similar ultrastructural changes were observed in cancer cells incubated with CPT6(campthothecin-20(s)-*O*-(2-pyrazul-l) aceticester [32] and polyamidoamine/flag-apoptin [33]. Additionally, in both 1301 (present study) and HL-60 [31] cells, the membranes were damaged. Alterations such as loss of plasma membrane integrity, swelling of cytoplasm, and disruption of organelles indicative of necrosis were described for HeLa cells incubated with gold nanoparticles (AuNPs) [30]. In contrast, CRD27 induced more marked ultrastructural changes in 1301 than in HL-60. While treatment of 1301 cells with CRD27 caused nuclear envelope disorganization when the lamina was separated from chromatin, and multi-vesicular and lamellar bodies and intramitochondrial vacuoles were formed, multi-vesicular, lamellar bodies, and secondary lysosomes appeared in HL-60 cells [31].

Ruthenium can react with DNA and damage it [34]. This effect has been observed with different ruthenium compounds [24,25]; hence, we examined the ability of metallodendrimers CDR13 and CDR27 to damage DNA in 1301 cells by comet assays. The data show that CRD13 exerted a stronger effect than CRD27, again consistent with the effects of these metallodendrimers on HL-60 cells [31].

Oxidative stress is related to decreased mitochondrial potential and can indicate an early stage of apoptosis [25]. In this study, both CRD13 and CRD27 increased the ROS level in 1301 cells, in contrast to Hippo-18 cells treated with carbosilane dendrimers [27]. When the carbosilane system was functionalized with ruthenium complexes, the ROS level in MCF-7 cells increased significantly [25].The metallodendrimers CRD13 and CDR27 altered the mitochondrial membrane potential in 1301 cells: CRD13 increased it, while CRD27 decreased it at lower concentration but increased it rapidly at higher concentration. In contrast, in MCF-7 cells, diruthenium-1 induced hyperpolarization [25]; carbosilane dendrimers did not affect the mitochondrial potential in Hippo-18 cells [27].

One mechanism of cellular death, apoptosis, can be regulated by Bcl-2 family members; hence, we analyzed the expression of pro- and anti-apoptotic proteins. In 1301 cells incubated with CRD13 at the lower concentration, the expression of Mcl-1 and BAX decreased. It was previously observed that the expression of anti-apoptotic proteins including Mcl-1 increases in tumor cells [34]. Several studies have shown that upregulation of Mcl-1is a key factor in the development of resistance in several tumor types [7,8,35,36]. Our results could therefore indicate the initiation of apoptosis [34]. Moreover, the expression of the apoptotic activator Bax protein decreased. Its activity reduces the membrane potential and the release of cytochrome C [34].

The increased APAF 1 expression reflects the beginning of the programmed cell death process [37], while increased Bcl-2 expression can inhibit apoptosis and cytochrome C release.

Incubation of 1301 cells with 5μmol/L CRD27 increased the expression of TNFα, APAF1, BAX, BID, and Mcl-1. The increase in Mcl-1suggests inhibition of apoptosis. High levels of BID are responsible for creating mitochondrial pores. TNFα and APAF1 initiate apoptosis. CRD treatment led to similar effects in HL-60 cells, suggesting that CRDs cause apoptosis and necroptosis in leukemia cells [31]. The results presented in this study seem to support this hypothesis.

## 4. Materials and Methods

### 4.1. Ruthenium-Terminated Carbosilane Dendrimers (CRD)

**Figure 6:**
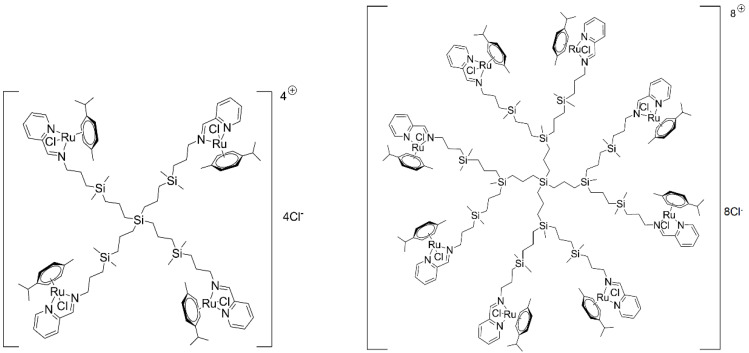
The structure and molecular weight of the 1st (CDR13) and 2nd (CRD27) generations of cationic carbosilane ruthenium (II) dendrimers.

The chemical structures of 1st and 2nd generation carbosilane metallodendrimers, based on arene ruthenium (II) complexes (CRD13 and CRD27) coordinated to iminopyridine surface groups, are illustrated in Figure 6. The main characteristics and synthesis of the dendrimers were described previously [11,15].

### 4.2. Cell Viability

The 1301 cell line (human ALL) was acquired from the ATCC Company, (Middlesex, UK). The cells were kept on plastic tissue culture flasks and plates (Falcon (Corning, NY, USA)) in RPMI-1640 (Gibco (Carlsbad, CA, USA)) with 10% heat-inactivated FBS (HyClone (Loga, UT, USA)) and 1% antibiotic mixture at 37 °C in a humidified atmosphere containing 5% CO_2_/95% O_2_. To assess the cytotoxicity of the CRDs, the cells were seeded onto 96-well plates at 1 × 10^5^ cells/mL. Their viability after 24 h incubation with the dendrimers was estimated by the Alamar Blue test and calculated from the formula Equation (1):

% viability = (A/Ac) × 100%(1)

where A is the absorbance of the sample, and Ac is the absorbance of the control cells. The results are presented as means from three independent experiments.

### 4.3. Determination of Reactive Oxygen Species (ROS) Levels

The 1301 cells were grown on 96-well plates at 1 × 10^5^ cells/mL in RPMI with 10% FBS and 1% antibiotics. They were incubated at 37 °C in a humidified 5% CO_2_/95% O_2_ atmosphere and were treated with dendrimers at 0.5–5.0 µmol/L concentrations. After the treatment, 2.5 µmol/L H_2_DCF-DA in PBS was added to the cell suspension and incubated for 20 min. Subsequently, fluorescence was measured using a BioTek Synergy HTX microplate spectrophotometer at λ_ex_ = 485 nm and λ_em_ = 528 nm. The ROS level was recorded as percentage vs. control. The results are means from three independent experiments.

### 4.4. Mitochondrial Transmembrane Potential

The 1301 cells were grown on 96-well plates at 1 × 10^5^ cells/mL in RPMI with 10% FBS and 1% antibiotics and incubated at 37 °C in a humidified 5% CO_2_/95% O_2_ atmosphere. They were treated with dendrimers at 0.5–5.0 µmol/L concentrations for 0.5, 3, 24, and 48 h. Next, a fluorescent dye, JC-1 (5.5′.6.6′-tetrachloro-1.1′.3.3′-tetraethylbenzimidazol-carbocyanine iodide) at 5 µmol/L was added to each well [38]. After 20 min incubation in the dark, measurements (ΔΨm) were made using a Fluoroscan Ascent FL microplate reader. The filters used were suitable for measuring fluorescence from monomers (l_ex_ = 485 nm, l_em_ = 538 nm) and dimers (λ_ex_ = 530 nm, λ_em_ = 590 nm). The fluorescence factor was calculated from the formula Equation (2):

(2) $$\Psi m=\frac{F_{D}}{F_{M}}$$

where Ψ*m* = mitochondrial potential, directly proportional to the fluorescence factor; *F_D_* = dimer fluorescence; *F_M_* = monomer fluorescence. The results were presented as mean ± SD (*n* = 3).

### 4.5. Comet Assay

The comet assay was performed under alkaline conditions following the procedure of Singh [39]. The 1301 cells were suspended in 0.75% low melting point agarose in PBS, pH = 7.4, and the suspension (5 × 10^−2^ mL) was spread on microscope slides precoated with 1% normal melting agarose. The slides were washed with 2.5 M NaCl, 100 mM EDTA, 1% Triton X-100, 10% DMSO, 10 mM Tris (pH = 10) at 4 °C for 1 h and immersed in electrophoresis solution (300 mM NaOH, 1 mM EDTA, pH > 13) for 40 min. Electrophoresis was conducted at 0.73 V/cm and 300 mA for 30 min. The DNA was stained (in the dark) with DAPI (4 µg/mL). After staining, fifty cells from each slide were examined using an image analysis system (Nikon E200. Tokyo. Japan) attached to a COHU 4910 video camera (CohuInc. (San Diego, CA, USA)) equipped with a UV Lucia-Comet v. 4.51 source (Laboratory Imaging, Prague, Czech Republic). The amount of DNA in each comet tail was determined.

### 4.6. Transmission Electron Microscopy

Ultrastructural changes in the 1301 cells were monitored as follows. Cells at 1 × 10^4^ per culture dish were grown in RPMI-1640 (Gibco) with 10% heat-inactivated FBS (HyClone) at 37 °C in a humidified 5% CO_2_/95% O_2_ atmosphere and were incubated with 2.5 µmol/L CRD13 or 5 µmol/L CRD27 for 24 h. After incubation they were centrifuged for 10 min at 1500 rpm and rinsed twice with phosphate-buffered saline (PBS) and then fixed with 2.5% glutaraldehyde in 0.1 M PBS, pH = 7.2, for 2 h. Subsequently, they were dispersed in 1.5% agarose and washed three times with the same buffer. They were post-fixed in 1% osmium tetraoxide for 2 h at 4 °C, dehydrated in ethanol and propylene oxide, and embedded in Epon–Spur’s resin mixture. Sample resin blocks were sectioned on an UltraCut E (Reichert Jung, Wetzlar, Germany) ultramicrotome with a diamond knife. Semi-thin sections were stained with 1% toluidine blue. Changes of cell morphology were examined under a Nicon Eclipse 50i light microscope using Coolview software. Ultrathin sections (70–80 nm) were placed on formvar-coated 300 mesh nickel grids and stained with uranyl acetate and lead citrate [40]. Cell ultrastructure was examined using a JEM 1010 transmission electron microscope (JEOL, (Akishima, Japan)) at 80 kV.

### 4.7. RNA Isolation, Reverse Transcription and Quantitative Real-Time PCR

RNA was isolated from 1301 cells using the commercially available Total RNA Mini Kit (A&A Biotechnology, (Gdynia, Poland)). The quality and quantity of RNA were estimated spectrophotometrically with a Bio-Photometer Plus (Eppendorf, (Hamburg, Germany)). A High-Capacity cDNA Reverse Transcription Kit (Applied Biosystems, (Waltham, MA, USA)) was used for reverse transcription in accordance with the manufacturer’s protocol. Total RNA (1 μg) was used in cDNA synthesis with the following incubation steps: 25 °C for 10 min, 37 °C for 120 min, and 85 °C for 5 min. The mRNA was quantified using real-time PCR with KiCqStart^®^ Primers (Sigma Aldrich, (Schnelldorf, Germany)). The reaction mixture consisted of cDNA, KiCqStart^®^ forward and reverse primers, PowerUpTM SYBRTM Green Master Mix (Thermo Fisher Scientific, (Waltham, MA, USA)) and RNase-free water in a volume of 10 μL. The cycle parameters were as follows: UDG activation at 50 °C for 2 min and Dual-LockTM DNA polymerase at 95 °C for 2 min, followed by 40 cycles of sequential incubations at 95 °C for 15 s, 59 °C for 15 s, and at 72 °C for 1 min. The results were normalized to the expression of HPRT1 (hypoxanthine phosphoribosyltransferase 1). All experiments were performedin triplicate. Master Realplex4s (Eppendorf) was used for real-time PCR. The fluorescent dye emission was a function of the cycle number. The initial amount of sample was recorded as a C_t_ parameter. The C_t_ value corresponded to the threshold cycle number at which PCR amplification reached a significant threshold. The relative expression level was calculated as 2^−∆∆Ct^ × 1000. The results are expressed as the number of mRNA copies examined per 1000 copies of HPRT1 mRNA.

### 4.8. Statistical Analysis

The results were analyzed statistically using a *t*-test (if the distribution was normal) or a Mann–Whitney Rank Sum Test (if the distribution was not normal). An ANOVA test was used to assess the significance of differences between particular dendrimers. When there were differences, further analysis was performed using Luke’s method. The threshold for significance was chosen at *p* = 0.05.

## 5. Conclusions

Carbosilane ruthenium dendrimers were selectively cytotoxic towards cancer but not to normal cells. This effect can be explained by the ruthenium atoms on the dendrimer surfaces. Both dendrimers examined induced programmed cell death in the 1301 cell line by apoptosis or necroptosis. However, the first generation of dendrimer was more toxic than the second generation. In the light of these results, ruthenium dendrimers seem applicable for drug delivery purposes in anti-cancer therapy.

## Figures and Tables

**Table 1:** Inhibitor concentrations (IC_50_ ± SD (µmol/L)) resulting in 50% dendrimer-mediated reduction of 1301 cell viability.

	24 h	48 h
CRD13	3.80 ± 0.36	3.65 ± 2.90
CRD27	4.90 ± 1.50	3.78 ± 2.70

**Table 2:** Changes in reactive oxygen species (ROS) levels in 1301 cells under the influence of ruthenium dendrimers CRD13 and CRD27. The values are mean ± SD.

Incubation Time	CRD13 Concentration (μmol/L)				CRD27 Concentration (μmol/L)			
	0.5	1	2.5	5	0.5	1	2.5	5
**0.5 h**	126.98 ± 8.80	127.27 ± 9.20	140.28 ± 10.51	140.81 ± 9.40	127.40 ± 9.52	126.87 ± 9.63	138.86 ± 8.48	141.19 ± 10.50
**3 h**	109.07 ± 5.00	109.70 ± 5.07	121.16 ± 0.99	127.89 ± 4.97	114.79 ± 2.88	116.36 ± 8.39	121.38 ± 6.34	122.18 ± 6.17
**24 h**	101.37 ± 2.54	100.68 ± 1.41	97.37 ± 2.08	95.49 ± 3.00	103.671 ± 1.73	99.47 ± 2.85	98.67 ± 1.89	94.02 ± 3.81
**48 h**	86.92 ± 8.43	90.64 ± 5.09	89.92 ± 7.65	93.47 ± 5.76	94.69 ± 4.48	89.28 ± 5.38	87.78 ± 3.44	88.08 ± 5.32

**Table 3:** Changes in mitochondrial potential (ΔΨm) in 1301 cells under the influence of ruthenium dendrimers CRD13 and CRD27. The values are mean ± SD.

Incubation Time	CRD13 Concentration (μmol/L)				CRD27 Concentration (μmol/L)			
	0.5	1	2.5	5	0.5	1	2.5	5
**0.5 h**	97.23 ± 3.28	96.43 ± 5.85	115.00 ± 3.55	122.145 ± 5.67	97.51 ± 2.53	99.62 ± 1.81	98.84 ± 1.44	109.09 ± 6.29
**3 h**	103.56 ± 5.56	109.63 ± 1.9	123.76 ± 1.60	140.56 ± 5.35	103.30 ± 4.43	109.79 ± 5.63	111.53 ± 13.53	138.58 ± 10.91
**24 h**	96.03 ± 6.65	96.63 ± 9.74	105.71 ± 14.06	120.40 ± 16.76	88.74 ± 9.48	92.79 ± 11.25	141.53 ± 16.90	176.4 ± 15.82
**48 h**	99.59 ± 3.28	97.92 ± 3.89	108.13 ± 6.15	119.94 ± 13.03	86.89 ± 5.72	84.02 ± 4.27	105.69 ± 10.14	120.51 ± 10.31
